# Galvanism

**Published:** 1810-04

**Authors:** M. M. Gay Lussac, M. Thenard


### GALVANISM.

*Extract from a Memoir presented to the Class of Mathematical and Physical Sciences of the National Institute of France.* By M. M. Gay Lussac and M. Thenard.

( From the Journal de Physique. )

M. M. GAY LUSSAC and THENARD, having proceeded to the decomposition of the boracic acid by the metal *potassium,* attempted in like manner the decomposition of the fluoric and muriatic acids, with the constituent principles of which we were as yet unacquainted. What was done with respect to the fluoric acid, and the principal results to which they have conducted us, as at present published by these philosophers, are here recorded.

Our first attention, say they, was to obtain the fluoric acid in a state of purity ; but as this acid does not exist but in a state of combination with lime, and that we have hitherto been unable to separate the two, without their entering into combination with other bodies, we have been under the necessity of making a considerable number of experiments, which has given the opportunity of ascertaining many facts, the most remarkable of which are as follow. When we brought potassium into contact with the fluoric acid gas, which becomes disengaged in an iron tube made red hot, containing fluate of lime and vitreous boracic acid, there thence result vapours of considerable density, similar to those formed from the combination of muriatic acid gas and ammoniacal gas. It yields them equally with every other gas, excepting with the muriatic acid gas, provided these have not been freed from moisture. But it alters the transparency of none else of them after they have been for some time in contact, whether it be with lime or with muriate of lime.

In the first case, when there is a production of dense vapours, the volume of the gas diminishes equally, and only a few hundredths of the centigrade thermometer. In the second case, when the gases preserve their transparency, their volume is by no means changed. We therefore conclude from hence, that the fluoric acid gas is an excellent test of the presence of moisture, or an hygrometric test in gases, and that all gases contain moisture, except the muriatic acid gas, the fluoric acid gas, and perhaps ammoniacal gas. It is hence, that in exposing the the muriatic and fluoric acid gases to a degree of cold of 15° or 19°, nothing is deposited, excepting a few traces of liquid; whilst on exposing the sulphureous acid gas, carbonic acid gas, &c. at the same degree of cold, it suddenly deposits the same quantity of aqueous moisture.

The dense vapours produced by the fluoric acid, in gases which contain water sensible to the hygrometer, betray, with respect to it, a great affinity for such water, so that it would not be exaggerating too much to say, that it would absorb more muriatic acid, and probably more than 2000 times its volume. When water is thus saturated, it is limpid, fuming, and more caustic. We obtain from it, by means of heat, more than one-fifth part of what it contains, and whatever methods are afterwards taken, it is not possible to obtain a greater proportion. It therefore resembles the concentrated sulphuric acid, and has both its causticity and aspect; like that, it does not enter into a state of ebullition, except at a much higher temperature than that of boiling water, and it is entirely condensed in *striæ,* although it may probably contain 600 times its volume of gas. Is it not extremely probable after this, if not even demonstrated, that the sulphuric and nitric acids should be gaseous if pure, and that they do not owe the liquid state in which they appear to any thing but to the water which they contain?

Although our fluoric acid gas has a great affinity for water, and that it contains none of it because it is derived from substances perfectly free from moisture, nevertheless, we neither know how to dissolve, nor how to render gaseous the minutest portion. We have put into contact for many hours over mercury, a litre of fluoric acid gas with one drop of water, and this drop, far from disappearing, augmented in volume. It is thus therefore proved, that this gas could contain water in no other way, neither as sensible to the hygrometer, nor in a state of combination.

Ammoniacal gas is precisely in the same situation, at least with respect to the water of combination. But this is by no means the same with respect to the muriatic acid gas; in truth, it contains no water sensible to the hygrometer, but it contains water in a state of intimate union, as was first shown by M. M. Henry and Bertholett. We have even obtained, by causing muriatic acid gas at a gentle heat to pass over litharge fused, and reduced to a gross powder, this water, and caused it to collect, which formed about one-fourth of its weight, according to the experiments periments which we have made upon the direct combination of a certain quantity of this acid gas with an excess of the oxyde of silver.

The other gases do by no means exhibit with water the same appearance as the preceding. None of them contain water in a state of combination, but all contain it, as indicated by the hygrometer. It follows then, from hence, that the fluoric and ammoniacal gasses neither contain water in a state sensible to the hygrometer, nor in a state of combination;* that the muriatic acid gas does not contain water, either in a state sensible to the hygrometer, or in a state of combination, and that all the other gasses simply contain water in a state sensible to the hygrometer.

What is more striking in these results is, to see that the muriatic gas contains water, and that the fluoric and ammoniacal gasses contain none whatever, and besides, to observe that the muriatic acid gas contains it in such proportion, that if it were entirely decomposed by a metal, all the acid would be absorbed by the oxyde, and become converted into a metallic muriate; it is the same, we may be assured, on passing the muriatic acid gradually, and in succession, through several gun-barrels made red-hot, and filled with iron turnings.

The more we reflect upon these phenomena, the more difficult it seems to be to account for them. Is it not possible that if oxygen and hydrogen be two constituents of the muriatic acid, that they may not be so in the form of water, but that they merely form it at the moment when this acid enters into combination with other bodies, so that in the muriates it would be in any other state than that of gas? However this be, one thing is certain, that all the muriates not decomposable by fire, and which contain little or no water, cannot be decomposed even at a very high temperature, either by the vitreous phosphate of lime, or the boracic acid, so that with respect to the muriates, their acid remains united with considerable force; and that if the sulphuric acid was itself deprived of water, it is more than probable that it could not itself decompose them. But let us not dwell too long on this hypothesis,

* It is certain, according to the experiments of Berthollet, jun. that ammoniacal gas does not contain water in a state of combination; but Gay Lussac and Thenard do not venture to assure us that it is not contained in a state perceptible by the hydrometer.

pothesis, but proceed to enquire into the properties of fluoric acid gas. We have already considered its physical properties, its action upon air, and upon the other gasses as well as water; let us now enquire into its action upon vegetables, which it acts upon with at least as great a force as the sulphuric acid, and appears like this acid to act upon them, in consequence of producing water and carbon; it also readily converts alcohol into good æther, a circumstance which we purpose more particularly to attend to, and it immediately blackens very dry paper exposed to its vapours, in consequence of the water formed and absorbed.

Every thing therefore proves that this fluoric acid gas is a perfect acid, not yielding in energy to any other with which we are acquainted, and that even in point of causticity it is equal to the concentrated sulphuric acid itself; and still it has no action upon glass. Until now we had considered it as being pure, but conceiving that it contained some substance which prevented its re-action upon silex, it readily occurred to us, that it held in solution a considerable portion of the boracic acid.

The fluoric acid obtained from the decomposition of fluate of lime by the boracic acid not being pure, we have attempted its decomposition by the acid phosphate of lime; we have been able only to obtain a small quantity, and the little which we have obtained, contained in the first place a small quantity of silex derived from the fluate of lime we employed, and in the last place a certain quantity of acid phosphate of lime itself. What is remarkable in this operation is, that when we employ siliceous fluate of lime, the decomposition of the salt is very rapid, in consequence of the action of the silex upon the fluoric acid, whence proceeds a considerable quantity of silicated fluoric acid gas.

Considering then that the fluoric gas proceeding from the fluate of lime and boracic acid contains no water, and that it is not capable of holding it in solution, we have conceived, against common opinion, that it is actually the case with such as is prepared in leaden vessels, by means of the concentrated sulphuric acid.

But instead of attaining, after this manner, this acid in a state of gas, we have obtained it in a liquid state, and possessed of the following properties. It exhales through the air a thick vapour, and in contact with water becomes hot, and even proceeds to the point of ebullition; it scarcely comes into contact with the glass but it erodes it; becomes becomes considerably heated, and finally, becomes converted to a silicated gas. Of all its properties, that of its action on the skin is the most remarkable. It scarcely comes into contact with it, before the organization is destroyed. A white speck is immediately perceived, and a considerable degree of pain succeeds. The parts adjacent to the point touched, speedily become white, and very painful, and in no long time after, a blister is formed, the sides of which have a white skin, very broad, and filled with pus.

How small soever be the quantity of acid, these phænomena equally occur; the developement of it is slowly made, and it is sometimes from seven to eight hours after it comes in contact, that it becomes observable; and although the burn be sufficient to produce considerable pain, to deprive one of sleep, and even excite fever; the effects of these kinds of burns are arrested, as we know by our own experience, by immediately applying after the accident, a weak solution of caustic potash, which we also know to be an excellent remedy for common burns.

We readily foresee that we are not to neglect to bring so active a liquid into contact with the metal obtained from potash. This experiment was performed in a tube of copper; we introduced a piece about the size of a hazel nut, of this metal, into a small quantity of the liquid potash, when a lively detonation instantly followed with considerable disengagement of light and heat.

When the metal potassium is thrown into liquid fluoric acid in a concentrated state, there is instantly disengaged a considerable portion of light and caloric, attended by the most vivid detonation; but afterwards, wishing to ascertain the cause of this phænomenon, the acid was gently poured over the metal, when heat was simply elicited, and the products of the experiment collected; those products were hydrogen fluate of potash and water, therefore the active liquid was a combination of water and fluoric acid.

We perceive therefore, that this acid has a tendency to combine with all bodies, and that it forms with them solid liquid or gaseous compounds, in proportion as it preserves its elasticity or expansive force; it is the only acid which has this property; and this circumstance alone is a proof that it is of all others the most active.

As we have no method of obtaining the fluoric acid pure, we cannot study it but in a state of combination with other bodies, only it is necessary to have it combined with with a particular body, according as we wish to obtain one result or another.

If it act by uniting with alcalies earthy metallic oxyds, it is necessary to prevent them from being acted upon by the silicated fluoric acid, or there would result tripple salts; it is thus, that in pouring ammoniac into the acid fluid of silex, we obtain a tripple salt, nearly insoluble, and in a considerable degree volatile; in like manner, by pouring the muriate of barytes into the acid fluate of silex, we obtain, after a short time, a crystaline insoluble precipitate in a considerable excess of nitric acid, which we may compound with the sulphate of barytes, and which is merely the fluate of silex and barytes.

But if instead of wishing to combine the fluoric acid with bodies, we would decompose its compounds by means of the metal potassium; it is then obvious, that we should not employ the fluoric acid in a liquid state, because the water contained in it, and which they would prefer, whether it be the fluoric gas holding in solution boracic acid, or rather the silicated fluoric gas, as the latter contains nothing combustible, it cannot lead us into an error, and cannot be prejudicial, except in consequence of the dispersion of the materials; thus it is, with respect to this gas, and especially with regard to the silicated fluoric acid gas, of which we have made use in our attempts to decompose the fluoric acid itself, and of which we proceed to give an account.

When we bring into contact at an ordinary temperature, the metal of potash with the silicated fluoric acid gas, it undergoes no sensible alteration, but simply becomes tarnished at its surface; but if we cause it to melt, it burns vividly, with intense light and heat; during this combustion there is a considerable absorption of fluoric acid, and a very slight disengagement of hydrogen gas, together with the production of a solid matter, the colour of which is a reddish brown. This matter, when treated with cold water, causes a disengagement of hydrogen gas, which does not however contain any portion of metal. If this matter be treated with cold water, and afterwards with warm hydrogen, it is disengaged in a much smaller quantity than in the former instance; and upon the whole, there is scarcely disengaged the third part of what was produced from the metal combined with water. If we connect the waters of ablution, and evaporate them, we shall obtain the fluate of potash with excess of alkali, and if we examine the residual after being well washed it is always of a reddish brown, and we find it possessed of the following properties: when thrown into a crucible of silver, and heated to a cherry red, it burns with great vividness, and the disengagement of a minute portion of acid gas; although previously insoluble in water, it now becomes soluble; the part which dissolves it is the fluate of potash, and what remains insoluble is the fluate of potash and silex. If, instead of making this experiment in a crucible, it be made in a small bell glass, in contact with oxygen gas, so as to become gradually heated, the ascension is much more vivid than when in contact with the atmospheric air; there is an absorption of a great quantity of oxygen, and the remaining gas, after combustion, is pure oxygen, with a small portion of fluoric acid gas; the product is solid, as in the preceding experiment, being formed of fluoric acid, lime, and silex.

It is evident, however, that during the combustion of potassium in fluoric acid gas, there is little or no hydrogen disengaged; we cannot attribute this combustion to the water, so that in this experiment, either the fluoric acid is decomposed, or at least, combined with the metal without oxydating it. These two hypotheses being the only ones we are able to advance, we shall discuss them in succession. If it were the metal which combined entirely with the fluoric acid, there would probably result from it a very immaliable compound, and which, with the acid of water, would yield as much hydrogen gas as the metal itself; whereas, we did not obtain more than one-third part of what we should in this case have done; therefore a combination of this kind militates against all the facts of every possible hypothesis, whether we consider the action of the fluoric acid upon metals and alkalies, or whether we consider the action of the metal potassium upon all the other acids; from hence, therefore, we conclude it to be probable, that the fluoric acid is itself decomposed. It should in consequence form, in this decomposition, a combination of the radical fluoric acid with the potash and silex. It appears, that when this radical is only combined with potash, it can decompose water like the phosphorets; but when it is combined with potash and silex, it is not at all decomposed; doubtless, in consequence of its being a triple salt, in such state of combination as to be insoluble.

However this may be, it is extremely easy to insure the combustion of the metal of potash with the fluoric acid gas.

The

The combustion being completed, and the vessel cooled, we withdraw, and detach the matter with a small spatula. This being effected, we can burn another quantity of the metal in this small capsule and glass, provided we cause to pass into it, the quantity of fluoric acid which had been absorbed in the preceding operation of combustion. We may in like manner make a third and fourth combustion; nothing prevents us, as we can always refill the glass to the same altitude with the fluoric acid gas, and can readily, at our pleasure, obtain the metal, by strictly attending to the process which we have laid down. We should however, add, with respect to this kind of experiment, to succeed compleatly, it is proper to absorb the unctuous matter floating on the surface of the metal by blotting paper, otherwise, it will spontaneously decompose, and yield a small proportion of hydrogen and carbon. In truth, it is with difficulty we can avoid this inconvenience; and whatever pains are taken, we shall always find a small portion of oil interposed between the metallic molecules, but its quantity is so small that it may be overlooked, and still no error be observable in the results. It is upon this oil that the property of the metals of potash and soda depend, of rendering lime water turbid.

